# Asymmetric hemisphere activation in tenderness: evidence from EEG signals

**DOI:** 10.1038/s41598-018-26133-w

**Published:** 2018-05-23

**Authors:** Guozhen Zhao, Yulin Zhang, Yan Ge, Yan Zheng, Xianghong Sun, Kan Zhang

**Affiliations:** 1 CAS Key Laboratory of Behavioral Science, Institute of Psychology, Beijing, China; 2 Department of Psychology, University of Chinese Academy of Sciences, Beijing, China

## Abstract

Emotions are processed asymmetrically by the human brain. Frontal alpha asymmetry (FAA) as measured by electroencephalographic (EEG) power in the alpha band (8–13 Hz), is a sensitive indicator of asymmetric brain activity in the frontal cortex. The current study aimed to analyze the frontal EEG asymmetries in terms of valence and motivational direction. We presented 37 participants with three film excerpts that were selected from the standard emotional film database to elicit three target emotions: tenderness, anger, and neutrality. Participants’ self-reports on their induced emotional responses and EEG signals were recorded and analyzed. The results showed that individuals displayed lower alpha power in the left hemisphere than the right hemisphere when they were watching a tender film, indicating that tenderness was positive and related to approach motivation. In contrast, when watching an angry movie, participants showed higher alpha power in the left hemisphere than the right hemisphere, suggesting that anger was negative and associated with withdrawal motivation. These findings help to link positive and approach-motivated tenderness with greater left hemispheric activation and state-anger with greater right hemispheric activation through the analysis of FAA.

## Introduction

Emotion is defined as an affective state of human beings and animals arising as a response to the perception of an object or situation^1^. It plays an important role in interpersonal events and communications^2^. Emotions are a psycho-physiological process and people have different heart rates, blood pressure, peripheral vascular resistance responses, and brain activities in different emotional states^3,4^. Among these psycho-physiological responses, asymmetrical frontal cortical activity has gotten more attention in the past three decades^5,6^.

Frontal alpha asymmetry (FAA) is a typical indicator of asymmetric brain activity in the frontal cortex, which refers to asymmetrical anterior electroencephalographic (EEG) activity in the alpha band (typically in the 8–13 Hz)^7^. To be specific, alpha power is inversely related to regional brain activity, and decreased power values of the alpha band indicate an increase in cortical or hemispherical activation^8^. FAA has been proven to be correlated with emotions, but the direction is not consistent. Some studies mainly focused on the relationship between resting FAA (i.e., trait-related FAA) and emotions, that is, a participant was asked to rest when EEG was recorded^9^. Other studies addressed emotional stimulation state-related FAA, in which EEG was recorded when a participant’s emotional states were elicited. Primarily, there are two predominant neurophysiological models accounting for the association between this alpha asymmetric pattern and emotions^10^.

The first one is called the valence model. The valence of emotion refers to the hedonic tone of the subjectively experienced emotions, ranging from negative or unpleasant emotions (such as sadness, fear, disgust, anger) to positive or pleasant emotions (such as happy, joy, amusement, contentment)^11^. According to the valence dimension, all emotions can be labeled as negative emotions or positive emotions^12^. From this perspective, greater left hemisphere activity (lower alpha power) is associated with positive emotion, whereas greater right hemisphere activity is associated with negative emotion^13^. Many researchers have supported this theory. From the recording of EEG during a resting period, greater left frontal activity was related to trait-like positive affects, whereas greater right frontal activity was related to trait-like negative affects^14,15^. Resting FAA is also associated with affective reactions. For example, Tomarken and his colleagues recorded resting EEG activity and extracted alpha power asymmetry from female adults. The authors found that people demonstrating stable and extreme relative left hemispherical activation reported increased generalized positive emotion and decreased generalized negative emotion compared to those who demonstrated stable and extreme relative right hemispherical activation^16^. As for state-related FAA, studies suggested that situational manipulations of positive and negative emotional states were associated with different asymmetric frontal activities. Wexler, *et al*.^17^ found that positive compound words produced relatively low activity in the right hemisphere and negative compound words produced relatively low activity in the left hemisphere without conscious awareness. Research has also found that voluntary facial expressions of fear were more likely to elicit less left frontal activity than controlled expressions^18^. Kline, *et al*.^19^ found that pleasant odors significantly induced greater left-hemispheric activity. Users revealed a stronger activation in the left hemisphere when watching pleasant advertisings and, conversely, a stronger activation in the left hemisphere associated with unpleasant ads^20^. Some studies also provided evidence of a causal relationship between FAA and valence using neurofeedback and unilateral hand contractions to manipulate FAA^21–23^.

Although the valence model has raised great support, some researchers argued that past work might confound valence with motivational direction^13^. According to most contemporary theories of emotion, positive emotion often correlates to approach-related motivation with more left frontalbrain activity, whereas negative emotion correlates to withdrawal-related motivation with more right frontal brain activity^24^. Thus, the second model, the motivational direction model or the approach-withdrawal model, is proposed. The approach-withdrawal motivational directionis defined as action tendencies directed at moving toward or running away^25,26^. This model states that FAA is linked to larger left-hemispheric activation during approach-related motivation but larger right-hemispheric activation during withdrawal-related motivation^15,16,27^. That is, emotions with approach motivational tendencies are linked to a higher left frontal activity, whereas emotions with withdrawal motivational tendencies are linked to a higher right frontal activity. Whether the relationship between a greater left frontal activity at the resting baseline and trait approach motivation measured by behavioral inhibition/activation system (BIS/BAS)^28^ actually exists is controversial^29^. However, extensive research has solidified the relationship between state-related FAA and motivational directions by manipulating situational motivational directions. A study in 2014 selected approach-positive, approach-negative, and withdrawal-negative pictures to test whether frontal asymmetry is related to motivation or affective valence. The authors found that greater left-frontal activation was associated with both positive and negative approach-motivated, which proved that asymmetric hemisphere activation was related to motivational direction, rather than affective valence^13^. Prause, *et al*.^30^ presented a neutral and a sexually motivating film to participants and recorded their EEG activities while watching. Greater alpha power in the left hemisphere during the sexually motivating film (i.e., approach motivation) was observed. A recent study used erotic pictures to elicit a higher level of approach motivation and FAA was recorded with an event-related design. Significant alpha asymmetries were found when erotic pictures were presented compared to pictures in controls^31^. In addition to healthy pariticipant, the results from patients with emotional disorders also support the notion that left frontal regions are involved in approach-related emotions and right frontal regions are involved in withdrawal-related emotions^32^.

Anger as a typical negatively valenced emotion has been reported to evoke behavioral tendencies of approach and link to approach motivation^33^. Several studies found that higher scores on a trait anger scale were associated with higher resting levels of left frontal activity and lower right frontal activity^34,35^. Similar results were also found in state-induced anger^24,36^. However, Harmon-Jones, *et al*.^5^ compared a situation in which individuals believed that they could engage in behaviors to ameliorate an anger-inducing event with a situation where they could do nothing, suggesting that the ability to react may moderate the relationship between anger and relative left frontal activity. It is important to note that some anger contexts are related to withdrawal tendencies and relative right frontal cortical activity^37^. Anger can be associated with withdrawal tendencies when individuals are inhibited from acting on their anger and they may ruminate about it^29^.

Tenderness is labeled as a positive emotion, which is different from anger on the valence dimension^38^. It is described as an expansive “warm-and-fuzzy” feeling, which is often elicited by the delicate and defenseless^39,40^. Considerable studies have concentrated on typical positive emotions such as happiness and joy^41,42^. However, little research has focused on this overlooked affiliative emotion, tenderness, which is associated with tender and warmhearted feelings. Tenderness is conceptualized as a momentary experience corresponding to love as caregiving^43^. That is, when you look in the large eyes of an infant or puppy, you will experience feelings of tenderness. Some evidence has noted that tenderness is related to an appraisal of vulnerability, such as observing an animal, an infant, or a child^44^. Consequently, it is different from another empathic emotion named sympathy, which is elicited by a current need rather than vulnerability^45,46^. Although tenderness is considered to be closely related to love and joy, it has been verified as a basic emotion with distinctive subjective experience^43^.

As a basic emotion, tenderness showed a different physiological and expressive pattern from other emotional categories in past research. For example, joy, anger, sadness, fear, and sexual arousal increased an individual’s heart rates, whereas tenderness decreased heart rates. The postural and facial expressions of tenderness could be effectively distinguished from other emotional categories^47^, because tenderness was associated with a specific breathing pattern. From the results of the functional magnetic resonance imaging (fMRI), tenderness activated the septohypothalamic area and the frontopolar cortex^48^, and humans were able to voluntarily modulate the activity of brain regions related to feelings of tenderness^49^. However, little research has been conducted to provide evidence for the relationship between tenderness and frontal alpha asymmetry using EEG.

Tenderness is also labeled as an affiliative emotion compared with non-affiliative emotions (e.g., anger) based on the frontopolar-septohypothalamic network^50^. However, the difference between tenderness and anger in terms of motivational direction has not been proven yet. Because tenderness can be regarded as the impulse toward caregiving behaviors, such as parental care and anticipation of future needs^51^, it is hypothesized that tenderness is associated with approach tendencies. Additionally, different emotional materials have been used to examine the relationships between FAA and emotions, including words, pictures, sounds, and facial expressions^9,52,53^. However, it is important to note that overwhelming majority of previous studies linked valence and motivational states with emotions using pictures such as stimulus from the International Affective Picture System (IAPS)^54^. Few studies have adopted film clips as emotion-eliciting stimuli. Because emotions are evoked by dynamic visual and auditory stimuli that are external to the individual, film clipsare more effective in eliciting strong emotional responses than pictures or sounds^55^. Moreover, scenes in film clips are usually authentic without deception, so film clips have a relatively high level of ecological validity^56^ and have the merit of being readily standardized as well.

Together, emotions are processed asymmetrically by the human brain and FAA is a sensitive indicator of emotional processing, both affectively and motivationally based on a valence model and a motivational direction model, respectively. According to the valence model, anger is a typical negative emotion related to right frontal activity whereas tenderness is a basic positive emotion related to left frontal activity. According to the motivational directional model, movie-induced angry state during which participants never expect any opportunity to retaliate and act on their anger may be asscoiated with withdrawal motivation, while tenderness may be associated with approach motivation reflected inrelative left frontal activity. Hence, there are two hypotheses in the present study. First, participants will display greater activation in the left hemisphere than in the right hemisphere (i.e., lower alpha power in the left hemisphere than in the right hemisphere) when they are watching a tender film clip. Second, participants will display greater activation in the right hemisphere than in the left hemisphere (i.e., lower alpha power in the right hemisphere than in the lefthemisphere) when they are watching an angry film clip.

## Method

### Participants

In this experiment, 43 healthy undergraduate or graduate students without neurological illness or psychiatric disorder were recruited. Six of them were excluded due to incomplete EEG recordings or equipment malfunctions, resulting in a valid sample of 37 (17 males and 20 females). Their average age was 23.95 years (SD = 1.56) ranging from 18 to 26. They are all right-handed and have normal or corrected-to-normal visions. All participants provided written informed consent and were paid for their participation. This study was approved by the Ethics Committee of Human Experimentation at the Institute of Psychology. All research activities were performed in accordance with relevant guidelines and regulations outlined in the approved protocol.

### Emotion elicitation materials

Three film clips were selected from the standard Chinese emotional film clips database^57^ to elicit three target emotions: tenderness (“*A Simple Life*” recalls the master’s happy childhood, 99 seconds in length), anger (“*City of Life and Death*” describes the scene of the Nanjing massacre, 73 seconds in length) and neutrality (In “Black Coal, Thin Ice” the hero takes his coat to a laundry, 65 seconds in length). According to our previous study, each film clip showed high validity based on the success index (i.e., a combination of hitting rate and target rating score) and successfully elicited the corresponding emotions.

### Self-assessment scales

Self-assessment scales were modified from a self-assessment manikin (SAM) (9-point Likert scale of arousal, valence, dominance, and liking) and 2-word differential emotions scale, (DES, tenderness, anger, 9-point Likert scale, 1 = “not at all”, 5 = “moderately”, 9 = “extremely”). Valence dimension ranged from “1-displeasure or unpleasant” to “9-pleasure or pleasant”. Arousal dimension ranged from “1-sleepiness” to “9-high arousal or excited”. Dominance dimension ranged from “1-controlled or being under control” to “9-controlling”. Liking dimension ranged from “1-like the scene and want to enter the scene” to “9-dislike the scene and want to escape from the scene”, which measures the subjective approach or withdrawal tendencies. The participants were instructed to rate all the scales according to their real feelings while watching the film clips instead of meeting the expected standards or their daily mood.

### Experimental procedure

**Figure 1:**
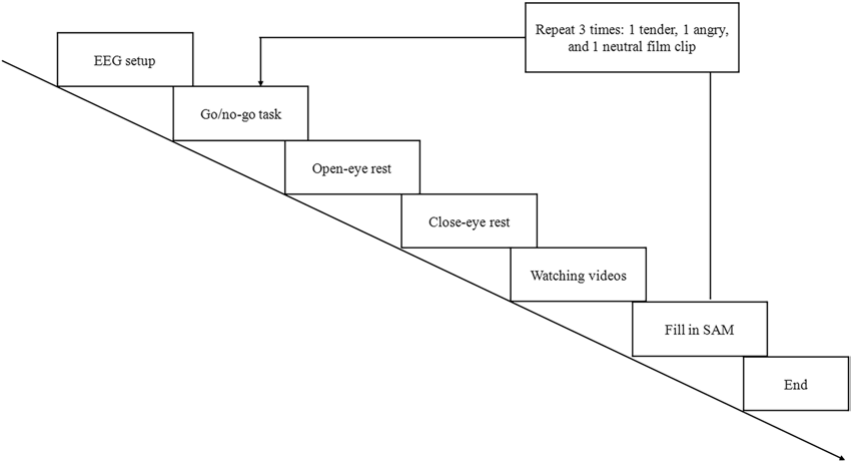
The timing diagram of the experiment.

Figure 1 shows the timing diagram of this experiment. Upon arrival, the participants provided informed consent and were given an introduction about the research work and each stage of the experiment to make sure that they knew the whole experimental procedure. They were required to hand over their cellphones, wash their hairs, and then were seated in a dimly lit, electrically shielded room and wore the EEG cap with the assistance of two experimenters. The experiment consisted of three trials. During each trial, a film clip was presented to the participants in a random order. Each trial started with a 60-s go/no-go task to keep participants in a neutral emotional state. Then, participants were asked to rest with their eyes open for 40 s and eyes closed for another 40 s to record baseline data. After resting, the film clips were presented to participants and they were instructed to watch the film and fill out the ratings earnestly. Each film clip was adjusted to the same resolution (720 × 576) without subtitles. Two speakers were used and the volumes of all the stimuli were set to an appropriate level to keep participants comfortable. Participants were seated, and their eyes were approximately 0.6 meters from the screen’s center. During the experiment, except for the break, participants were asked to keep their chin on the chin strap. EEG signals were recorded throughout the whole experiment.

### EEG acquisition

EEG signals were recorded using a NuAmps 40-channel monopolar DC amplifier system sampling at 1000 Hz with 22-bit resolution. 32 active silver-chloride electrodes (Ag/AgCl) were distributed using the 10/20internationally recognized placement system. A ground electrode was mounted at the center of the forehead and 4 cm above the nasion. To reject ocular artifacts, the electrooculogram (EOG) was recorded via four electrodes. Two horizontal electrodes were placed at the outer-canthus of both eyes and two vertical electrodes were placed above and below the left eye. All electrode impedances were kept below 5 kΩ.

We used the Neuroscan software 4.5 to process EEG data. EEG data were filtered with a 1–30 Hz bandpass. Then, we looked through a bunch of eye blinks (channel VEOG) and figured out a threshold that would catch the majority of them for each participant. All the blinks within the threshold we assigned were segmented into epochs (interval = −200–600 ms) and rejected in the set that contained more than 1 eye blink or appeared to deviate from the norm. The spatial singular value decomposition (SVD) was performed to create the ocular artifacts linear derivation (LDR) file which was subject-dependent and used to approximate the topographies for each component to be removed from the EEG raw data. After that, we went through and blocked off sections with aberrant signals by hand. The current source density (CSD) transformation was applied to artifact-free data using the CSD Matlab toolbox^58^, which is reference-free and shows excellent reliability as well as temporal stability^59,60^. Data were then transformed into a frequency domain by a short-term Fast Fourier transformation through a 2-s Hanning window with a 50% overlap between two consecutive windows to minimize data loss due to windowing. Power values within the alpha band (8–13 Hz) were averaged across windows for each resting period and each film clip. Finally, we normalized the average alpha power values to eliminate the confounding effects of the last film clip on the current one as well as individual differences in EEG signals. Specifically, the power of alpha band when watching each film clip and the corresponding resting period with eyes open were both log transformed. Then, the log transformed power values of watching videos minus the log transformed power values of the resting period with eyes open was measured, which was utilized as the final average power values^57,61^.

### Data analyses

A 3 × 2 × 2 repeated measures ANOVA was conducted for the average power spectral density of the alpha rhythm with Emotion Category (tenderness, anger, neutrality), Electrode (FP1/FP2, F3/F4) and Hemisphere (left, right) as three within-subjects variables. When an interaction or a main effect was significant, post-hoc comparisons or simple effect analyses were performed with a Bonferroni’s correction.

### Data availability statement

The datasets generated during and/or analysed during the current study are available from the corresponding author on reasonable request.

## Results

### Subjective ratings

The results of subjective ratings showed that both tender and angry movies successfully elicited target emotions with a hit rate of 100%. That is, each film’s anticipated target emotion of all 37 participants received a higher rating (at least 1 point) than the other two emotional categories. The average target rating score for tenderness was 7.22 (SD = 1.64), and anger was 7.70 (SD = 1.53). The neutral film elicited very low levels (less than 2 points on average score) of the other two target emotions with an average score of 1.49 (SD = 1.22) for tenderness and 1.14 for anger (SD = 0.35).

**Figure 2:**
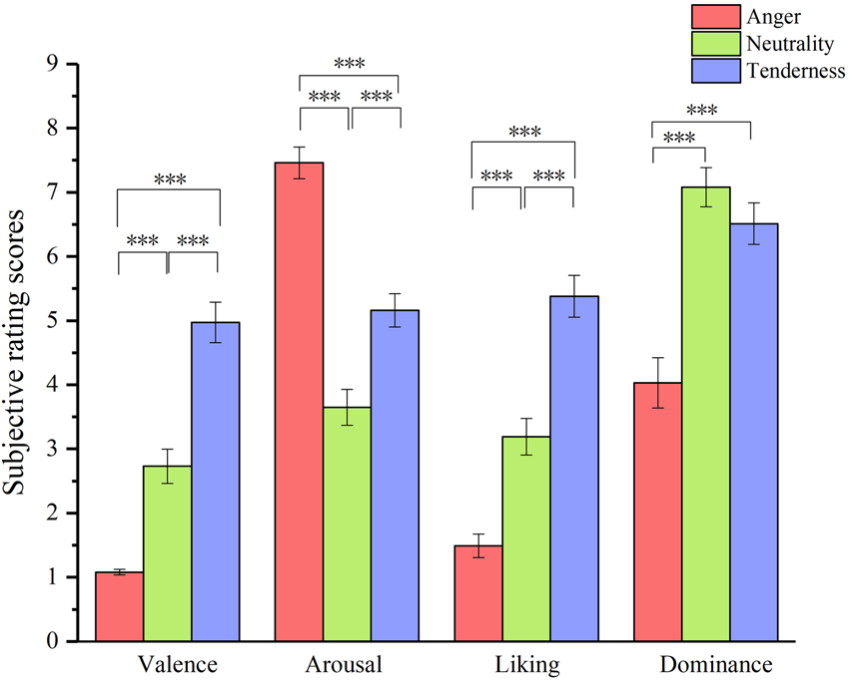
Subjective ratings of valence, arousal, liking and dominance in three emotional categories. Error bar represents ±1 standard error of the corresponding mean.

One-way repeated measures ANOVAs were conducted on the valence, arousal, liking, and dominance scores (see Fig. 2). The means and standard deviations for the subjective ratings of valence, arousal, liking and dominance in three emotional categories were presented in Table 1. The results showed a significant difference in the self-reported scores on valence among the three emotions (*F*(2, 72) = 89.49, *p* < 0.001, η_p_^2^ = 0.713). A post hoc analysis showed that the scores on the valence dimension were the highest for positive emotions, which were significantly higher than the scores for neutral and negative emotions (*ps* < 0.001). The higher score on the valence dimension represented the more positive characteristics of an emotion. Similarly, the results showed a significant difference in the self-reported scores on arousal among the three emotions (*F*(2, 72) = 82.04, *p* < 0.001, η_p_^2^ = 0.695). A post hoc analysis revealed that an angry film clip elicited a higher arousal state than a tender movie which elicited a higher arousal state than a neutral video (*ps* < 0.001). One-way repeated measures ANOVAs results also revealed significant differences in both liking (*F*(2, 72) = 77.41, *p* < 0.001, η_p_^2^ = 0.683) and dominance scores (*F*(1.629, 58.638) = 27.67, *p* < 0.001, η_p_^2^ = 0.435). A tender film clip received higher ratings on the liking dimension than an angry movie which received lower ratings than a neutral video (*ps* < 0.001). The tender film clip received significant higher ratings on the dominance dimension than an angry movie (*p* < 0.001).

**Table 1:** The means and standard deviations for the subjective assessment of valence, arousal, liking and dominance in three emotional categories.

	Valence	Arousal	Liking	Dominance
Tenderness	4.97 (1.92)	5.16 (1.59)	5.38 (1.99)	6.51 (1.97)
Anger	1.08 (0.28)	7.46 (1.50)	1.49 (1.12)	4.03 (2.40)
Neutrality	2.73 (1.63)	3.65 (1.70)	3.19 (1.73)	7.08 (1.86)

### Frontal alpha asymmetry

**Figure 3:**
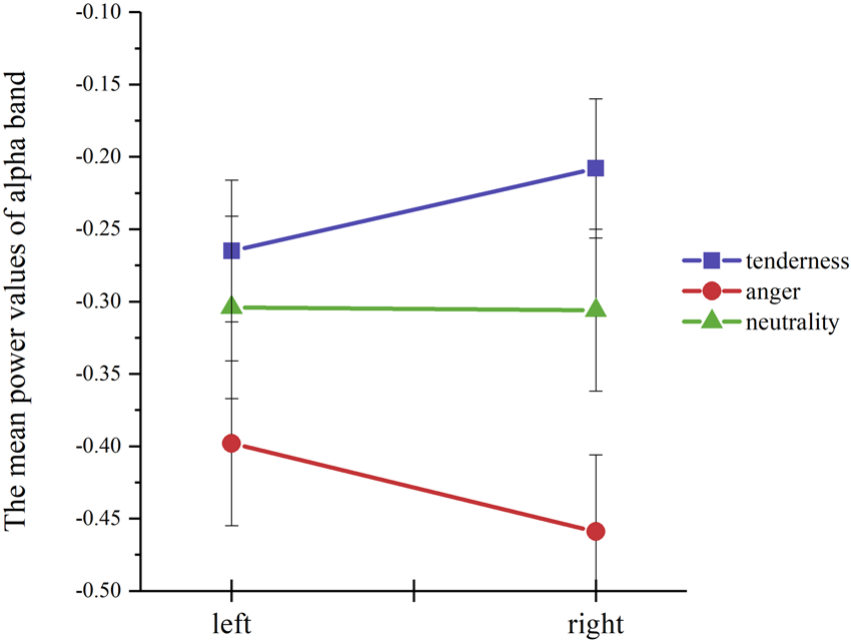
A significant emotion category × hemisphere interaction for the mean power values of the alpha band. Error bar represents ±1 standard error of the corresponding mean.

Results of 3 × 2 × 2 repeated measures ANOVA showed that the three-way interaction of emotion category × electrode × hemisphere was not significant for the mean power values of the alpha band (*F*(1.653, 57.841) = 1.728, *p* = 0.185, η_p_^2^ = 0.047). The two-way interaction of emotion category × hemisphere (*F*(2, 70) = 10.316, *p* < 0.001, η_p_^2^ = 0.228) was significant for this measure (see Fig. 3). The means and standard deviations for the mean power values of the alpha band were presented in Table 2. Simple effect analysis was conducted to assess the differences in the mean power values of the alpha band between the left and right hemisphere for each emotional category (see Table 3). Results revealed that participants displayed lower alpha power in the left hemisphere than the right hemisphere when they were watching a tender film clip (*p* < 0.01), indicating greater left frontal cortical activation in tenderness. In contrast, lower alpha power was observed in the right hemisphere than the left hemisphere when participants were watching an angry film clip (*p* < 0.01), indicating greater right frontal cortical activation in anger. No significant difference in the alpha power was found between the left and right hemisphere when a neutral movie was presented (*p* = 0.929).

**Table 2:** The means and standard deviations for the mean power values of the alpha band.

Electrode	Hemisphere	Emotion Category		
		Tenderness	Anger	Neutrality
FP1/FP2	Left hemisphere (FP1)	−0.24 (0.27)	−0.35 (0.34)	−0.31 (0.37)
	Right hemisphere (FP2)	−0.16 (0.32)	−0.42 (0.35)	−0.27 (0.33)
F3/F4	Left hemisphere (F3)	−0.28 (0.36)	−0.45 (0.39)	−0.32 (0.44)
	Right hemisphere (F4)	−0.26 (0.31)	−0.49 (0.33)	−0.34 (0.37)

**Table 3:** Pair-wise comparisons for the mean power values of the alpha band between the left and right hemisphere for each emotional category.

Emotion	Pair-wise comparisons	*p*-Value	*i*-*j* (95% CI)
Tenderness	Left (*i*) vs. Right (*j*)	0.010	−0.057 (−0.098, −0.015)
Anger	Left (*i*) vs. Right (*j*)	0.003	0.061 (0.023, 0.099)
Neutrality	Left (*i*) vs. Right (*j*)	*NS*	

Both main effects of emotion category (*F*(2, 70) = 4.643, *p* < 0.05, η_p_^2^ = 0.117) and electrode (*F*(1, 35) = 6.892, *p* < 0.05, η_p_^2^ = 0.165) were significant for the mean power values of the alpha band. Higher alpha power (lower cortical activation) was observed at FP1/FP2 electrode sites than F3/F4 (mean difference and 95% CI for difference: 0.069 (0.016, 0.122), *p* < 0.05). Moreover, the other two-way interactions of emotion category × electrode (*F*(1.640, 57.399) = 0.472, *p* = 0.654, η_p_^2^ = 0.012) or electrode × hemisphere (*F*(1, 35) = 0.864, *p* = 0.359, η_p_^2^ = 0.024) were not significant for this measure. No significant main effect of hemisphere was observed for this measure (*F*(1, 35) = 0.021, *p* = 0.885, η_p_^2^ = 0.001).

## Discussion

The present study examined the EEG patternsand tested FAA patterns in three emotional categories: tenderness, anger and neutrality. The results proved that individuals displayed lower alpha power in the left hemisphere than in the right hemisphere when they were watching a tender film. Combining the resluts of subjective assessments, it can be illustrated that tenderness was a positive emotion and might be related to approach motivation. By contrast, when participants watched an angry film, individuals displayed lower alpha power in the right hemisphere than in the left hemisphere. Results from subjective assessments proved that the anger emotion in the current study might be associated with withdrawal-related motivation.

In line with the previous findings^50^, the current study proved that tenderness was a typical positive emotion that was opposite to anger on the valence dimension. Additionally, we found that tenderness as a basic positive emotion was related to greater left frontal cortical activation and approach motivation. Tenderness is considered to be an empathic concern that is elicited by and congruent with the perceived welfare of someone in need^45^. It is evoked by the perception of vulnerability, which is a kind of need elicited by vulnerable targets viewed as comparatively weak and defenseless^40^. The vulnerability need describes a situation in which the target is seen vulnerable to future discrepancies even when there is no discrepancy between one’s current state and what one desires on one or more dimensions of wellbeing^45^. Imagine that you are seeing a beautiful baby sleeping peacefully, you can still feel the feeling of tenderness even when the baby has no current need. Understanding what causes tenderness is of vital importance, because research shows that tenderness has powerful motivational consequences. It is noted that empathic concern produces altruistic motivation and tenderness is believed to elicit an approach orientation toward others in need and to facilitate pro-social behavior in order to relieve the need^62^. Due to a view of human parental nurturance, empathic emotions such as tenderness are the embodiment of the cognitive generalization of parental nurturance, which plays the role of promoting the protection and care of offspring and young^63,64^. Consequently, tenderness has approach motivational consequences for subsequent helping intentions such as protecting and shielding the other from harm^46^. This approach motivation is associated with some long-range forms of assistance (such as training and education) as well as the monitoring of the target’s situation (such as monitoring potential threats and dangers)^65^. With regard to physiological patterns of tenderness, the current study provided evidence for this approach motivation by means of FAA. Although there is no immediate or subsequent altruistic behavior, the approach intentions can still be reflected in thegreater left frontal cortical activation (i.e., lower left alpha power) when individuals experience the feelings of tenderness.

Another interesting finding in this study was that film-elicited state anger might be related to the greater right frontal cortical activation (although not significant) and withdrawal tendencies. However, past research has demonstrated that anger was associated with approach motivation. It is suggested that anger does not always increase the left frontal activity and it is not always related to approach motivation. A few studies have provided support for this point of view. For example, 165 active soccer players were instructed to imagine that they had been preparing for a match for a long time and were looking forward to proving themselves but they were unfairly prevented from doing this by the coach. Participants imagined approaching the coach and starting to protest in the anger-approach group, whereas participants imagined backing out of the locker room and silently swearing at the coach in the anger-withdrawal group. The results showed that anger-approach and anger-withdrawal did not differ from each other in the left frontal activation although both conditions evoked anger effectively^6^. One explanation was that individuals still reported being angry but did not show an increase in the left frontal activity when they believed there was nothing they could do to rectify an angering situation^66^. Consequently, whether anger is linked to approach motivation or withdrawal motivation depends on the expression possibility, coping patterns and the characteristics of the angering situation. For instance, an avoidance and withdrawal motivation prompted by anger before an interracial interaction was found in two studies, which additionally revealed that this event-induced angry situation was linked to withdrawal motivation^37,67^. Similarly, in current study, participants reported significantly lower liking (greater withdrawal tendencies) of anger compared with tenderness, but FAA showed no significant difference between left and right hemispheres. For the subjective assessments, in the current study, the anger-eliciting film would induce such emotion with withdrawal tendencies when participants were instructed to keep silent and stay focused when watching the film. They had no chance to express their anger and they knew that they could not change the current situation and the history described in the clip, but could only keep their anger in and control their feelings, which leads to a feeling of frustration. For frontal activities, one possibility is that the frontal asymmetry stands for behavioral approach or avoidance motivation^68^. However, participants were instructed to keep their chins on the chin strap without any sort of relevant behavioral tendencies. Even if they felt angry but cannot show evident behavioral motivation. This manipulation limited the explanation of our results to some extent for testing the motivation direction theory, so some measurement of behaviors could be added in the future studies. Also, the current measurement of motivational direction through the “liking” dimension was indirect and may measure both motivational direction (e.g. want to enter the scene) and a component similar to valence (e.g. like the scene). A more direct way to measure state motivational direction should be considered in the future studies.

It is worth noting that the current experiment helps to improve the understanding of the frontal asymmetry of more basic positive emotions through FAA. Past research has concentrated on a few positive basic emotions associated with frontal asymmetry, such as happiness and joy^18^. Other research examined positive social emotion such as love^13^. However, love does not refer to an emotion but to a disposition to respond emotionally^69^, whereas tenderness as a basic social emotion corresponds to love as caregiving^43^. Thus, the present study provided a basis for using tenderness as a positive and approach-motivational target emotion in the study of frontal asymmetry. Additionally, the associations between emotions and FAA have been applied in marketing, design, and emotion-related products and user experiences^7^. FAA indices can be used as featuresin affective computing and emotion recognition as well, and the previous study has validated the possibility of FAA-based emotion classification^14^. Broadly, the current experiment helps to link positive and approach-motivated tenderness with the greater left hemispheric activation and film-elicited anger with the greater right hemispheric activation through the analysis of FAA.
